# Trust is the common denominator for COVID-19 vaccine acceptance: A literature review

**DOI:** 10.1016/j.jvacx.2022.100213

**Published:** 2022-09-29

**Authors:** Bipin Adhikari, Phaik Yeong Cheah, Lorenz von Seidlein

**Affiliations:** Mahidol Oxford Tropical Medicine Research Unit, Faculty of Tropical Medicine, Mahidol University, Bangkok, Thailand; Centre for Tropical Medicine and Global Health, Nuffield Department of Medicine, University of Oxford, Oxford, UK

**Keywords:** Trust, Vaccine, COVID-19, COVID-19 vaccine, Vaccine hesitancy, Willingness to take vaccine, Perceptions, Institutional trust

## Abstract

Vaccine hesitancy and refusal to be vaccinated are major reasons why mass vaccination strategies do not reach the intended coverage, even if adequate vaccine supply has been achieved. The main objective of this study is to explore the role and contribution of trust in public willingness to accept COVID-19 vaccinations. The study utilised a qualitative synthesis of literature around hesitancy, willingness to accept vaccination, and the role of trust. Data were extracted from the literature and first categorised using a deductive approach, and later analysed in QSR NVivo using a mix of deductive and inductive approaches. The impact of trust was mostly borne out in the willingness to accept a vaccine, but details on what trust is, how and why it affects willingness or lack of it, was not frequently reported. Three types of trust were identified: 1) Trust in the quality and safety of vaccines; 2) Institutional trust; and 3) Interpersonal trust in the professionals who communicate about and administer the vaccine. Trust in the vaccines’ quality and safety, and institutional affiliation significantly contributed towards willingness to be vaccinated. The bulk of the literature focused on how interpersonal trust and personal attributes of potential vaccinees affected the willingness to accept the vaccine. This complex relationship included a fragility of beliefs and perceptions at an individual level, with a bidirectional relationship to societal perceptions. Perceptions of vaccines had a predominant role in decision-making, in contrast to more science-based decision-making. Although globally, the perceptions and beliefs contributing to trust had commonalities and relevance, trust was often found to be dependent on factors embedded in local social, cultural, institutional, and individual attributes and experiences. Understanding different types of trust offers potential approaches to motivate undecided people to receive vaccine; and vaccine refusers to revisit their decisions.

## Highlights

• Vaccine hesitancy is a central barrier to ending the COVID-19 pandemic.
• Trust in the quality and safety of vaccines and the institutions dispensing the vaccines is critical for the willingness to be vaccinated.
• Trust in vaccines and institutions are subject to individual and societal perceptions built over time, and vary by social, cultural and historical context.
• Preconceived perceptions of COVID-19 vaccines played a more important role in decision-making than science-based information.
• Targeted and tailored approaches are essential to build trust in COVID-19 vaccines.

## 1 Introduction

Trust is a fundamental element of acceptance of public health interventions by the targeted population [1]. Yet what constitutes trust, the impact trust (or a lack thereof) can have in an intervention, and how different types of trust contribute to the uptake and acceptance of an intervention, is elusive [1], [2]. The basic attributes of a vaccine, such as the perceived safety and protection offered, contribute to the decision whether or not to accept the vaccine, but a large proportion of the population place their trust in the institutions who provide vaccines, without fully understanding the science behind them [3]. Even those who have access to the latest vaccine science have to take the final ‘leap of faith’ due to residual uncertainty [4].

Trust in the science is referred to as ‘epistemic trust’, that is, placing trust in the competence of a person, organisation or institution who promote science related knowledge or its product [5]. Trust in vaccines reflects epistemic trust which builds on 1. Trust in the vaccine as a product weighing on its safety and quality; 2. Institutional trust: where vaccine comes from (institutional affiliations, organizations and their reputations); and 3. Inter-personal trust: who recommends the vaccine (recommendations by health care workers, neighbours, relatives and peers) and the nature of the recommendations (positively convincing versus negatively convincing) [6]. Inter-personal trust is a relational notion that describes a voluntary relationship between two persons based on past interactions, reputation, and competence that build the current expectation [1], [7]. An interaction between a person and an institution yields an institutional trust that is built through years based on the knowledge, competence, and skills that the state, institution, or the health care workers bear [1], [3], [7]. A typical example is how patients are inclined to follow instructions when they trust their health care workers [8]. Personal attributes such as race, ethnicity and socio-demographic backgrounds also affect the perceptions related to the disease, protective effect of the vaccines ultimately affecting the trust towards the vaccine and the decision to accept vaccination. Default asymmetry in information, comprehensibility, and power between the vaccine providers and the vaccine recipients makes the person who has to make a decision regarding vaccination vulnerable as they have to invest some degree of faith in the trusted party [3], [6].

Vaccine hesitancy has been defined as a state of uncertainty in decision-making due to doubts about the benefits of vaccines, their safety and necessity; and is a transient stage where a candidate may weigh the risks versus benefits or more emotional aspects associated with vaccinations [6], [9]. Vaccine hesitancy is complex and context-specific, varies over time, place, and type of vaccines; and is affected by factors such as confidence in the vaccine, complacency, convenience, rumours, and emotions [10], [11]. A global survey conducted in 2021 in 19 countries found that around one-third of participants hesitated to take a COVID-19 vaccine, with acceptance of a vaccine ranging from 90 % in China to 55 % in Russia [12]. Much like public health interventions, acceptance and uptake of vaccines are dependent on whether populations place their trust in the vaccine itself (trust in the product), the institution that provides the vaccine (institutional trust), and the professionals who communicate and administer it (inter-personal trust) [3], [13]. In the wider literature, trust is also described as a relational notion, an intermediary element (or its absence) inherent in the outcome of an intervention [14]. The abstract nature of trust is often described in terms of its constructs such as trust in a vaccine as a product with a defined protective efficacy and duration of protection. While trust is a critical factor in vaccine-related decision making (vaccine refusal, hesitancy, or vaccine acceptance), the decision whether to be vaccinated or not may occur without a deliberate investment in trust (or lack thereof). For instance, a person may decide to be vaccinated when obliged by government regulations such as a vaccine mandate.

Despite the benefits of vaccines in preventing deaths and diseases, vaccine uptake has always been inconsistent. This is reflected in the COVID-19 vaccine roll-out [15], [16] and public responses, where the absence of trust has been recognised as a key inhibitor to uptake. Nonetheless, the relational nature of ‘trust’ and the factors affecting it are difficult to conceptualise and consolidate [17]. Larson et al have conceptualised that “vaccine trust” was dependent on the product, provider, and its institution, in addition to the broader local social, cultural and historical context [3]. Historical neglect or abuse from a government or health system in the United States, for example in the Tuskegee Syphilis study was seen as the main reason for distrust in vaccines among minority US populations [18]. Improvement in healthcare, mitigation of discrimination and strengthening of inclusion and diversity efforts may garner trust [19]. While there are factors and elements of trust related to vaccines in general, there are specific attributes unique to COVID-19 [20]. It is therefore critical to explore the relevance of trust in vaccines and COVID-19 vaccines specifically, to inform the tailoring of vaccination programmes for the current and future pandemics.

With the steady increase in production of COVID-19 vaccines, globally vaccine supplies have become less of a problem compared to the vaccine hesitancy [21]. In contrast to previous reviews reporting multitude of factors that contribute to vaccine hesitancy (acceptance), this review examines whether and how various factors contribute to epistemic trust towards vaccine. Historical vaccination programs, policies, and incentives to promote the uptake of vaccine (including the current COVID-19 vaccine programs) have time and again implied the need to build and sustain trust relationships between the public and vaccine for a sustainable solution [21], [22]. This review explores how trust is an essential element to promote vaccination programs.

Globally, elements and factors affecting trust towards COVID-19 vaccines are constantly evolving, and with it willingness to accept vaccinations [3], [13]. The lack of scientific literature related to trust around COVID-19 vaccines, combined with the sprouting of non-empirical research, warrants a synthesis of available evidence. This qualitative review draws from global literature around COVID-19 vaccine and is supplemented by historical and generic vaccine-related literature. The main objective of this review is to explore to what extent trust is the essential common denominator for COVID-19 vaccine acceptance.

## 2 Methods

For the qualitative literature review, three major databases (MEDLINE, Scopus, and Web of Science) were searched. The search strategy was built around two main concepts: COVID-19 vaccines and hesitancy (Supplementary data 1). Variants of terms around the main concepts were utilised to generate literature relevant for the review. The searches included literature published in English until 12th May 2021. This review utilizes a previously used method that blends systematic literature search and thematic (qualitative) synthesis of the findings; such a mix of methods can compensate for the narrow and prescribed methods outlined by a systematic review while at the same time allowing exploration of broader themes related to the research question [23].

**Fig. 1:**
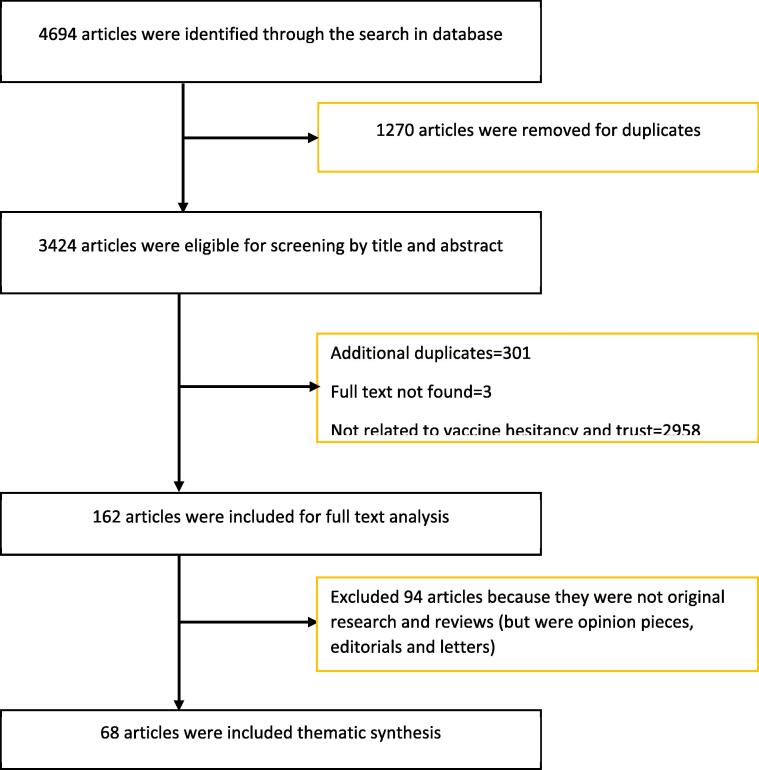
Flowchart showing systematic search of literature up to 12 May 2021. A total of 68 articles were included in the full text analysis of this review.

A total of 4,694 articles were identified through the search in the three databases (Fig. 1). After removing the duplicates in Endnote (Clarivate Analytics, London, United Kingdom), 3,424 articles were retained for title and abstract screening. A total of 3,262 articles were removed based on the screening by title and abstract, and 162 full text articles were included for full text analysis. A total of 68 original articles were finally included in the thematic synthesis (Supplementary data 2) based on the inclusion and exclusion criteria. Studies explaining trust around COVID-19 vaccines and the range of outcomes (vaccine refusal or acceptance) were explored to synthesize the themes in the review.

### 2.1 Inclusion criteria

1. Studies reporting trust around COVID-19 vaccines AND
2. Studies about COVID-19 vaccine hesitancy OR
3. Studies about willingness to accept COVID-19 vaccines.

### 2.2 Exclusion criteria

1. Studies that do not report sufficient details about COVID-19 vaccines and trust.
2. Non-original research such as opinion pieces and editorials.

### 2.3 Characteristics of the studies

Most studies (65/68) included in this review used quantitative methods, specifically survey questionnaire to assess the trust, vaccine hesitancy, acceptance, and refusal. 17/68 (25 %) of the studies were from the USA. Although few studies measured trust explicitly, most studies (61/68) measured the degree of likelihood to get vaccinated if COVID-19 vaccine was available – a proxy measure of trust in vaccine. Only a few studies (n = 2) specifically used the WHO SAGE vaccine hesitancy scale or another validated tool while most used one or two questions related to willingness to get vaccinated or intention to get vaccinated by COVID-19 vaccine. Because most studies were conducted during the COVID-19 pandemic, respondents were either surveyed online or through telephone and only in exceptional cases through face-to-face interviews. The lowest sample per study included was 100 and highest was 185,000, and most studies collected data during second, third and fourth quarter of 2020.

### 2.4 Thematic synthesis

Three major themes were categorised based on the previous literature [1], [3], [7]. Data were deductively analysed and extracted under each of the following themes: (1) Trust in vaccine (safety and quality related to vaccines); (2) Institutional trust; and (3) Interpersonal trust and/or personal attributes. In this review, three categories of trust related to vaccine were used as *a priori* themes because of its relevance to COVID-19 vaccine. Interpretations of authors were added to contextualise the extracted data where relevant. At first, data were extracted into tables under each of these three themes, followed by coding of the data in QSR NVivo (QSR International, Doncaster, Australia). Tabulated data were coded line by line (and sometimes by single terms). Although three categories of trust (themes) guided the broader data coding, statements or terms (as sub-themes) were coded multiple times under each theme as they were highly correlated. Thematic synthesis and exploration of literature continued until no new data/themes were identified. Additionally, descriptions around factors and underpinning mechanisms were scoped in the literature.

## 3 Results

### 3.1 Overview

Trust and its impact on vaccine hesitancy was reported in the majority of articles, but the elements of trust and their mechanisms were infrequently explained. Three major types of trust relevant for vaccine uptake were included and discussed in this review.

**Fig. 2:**
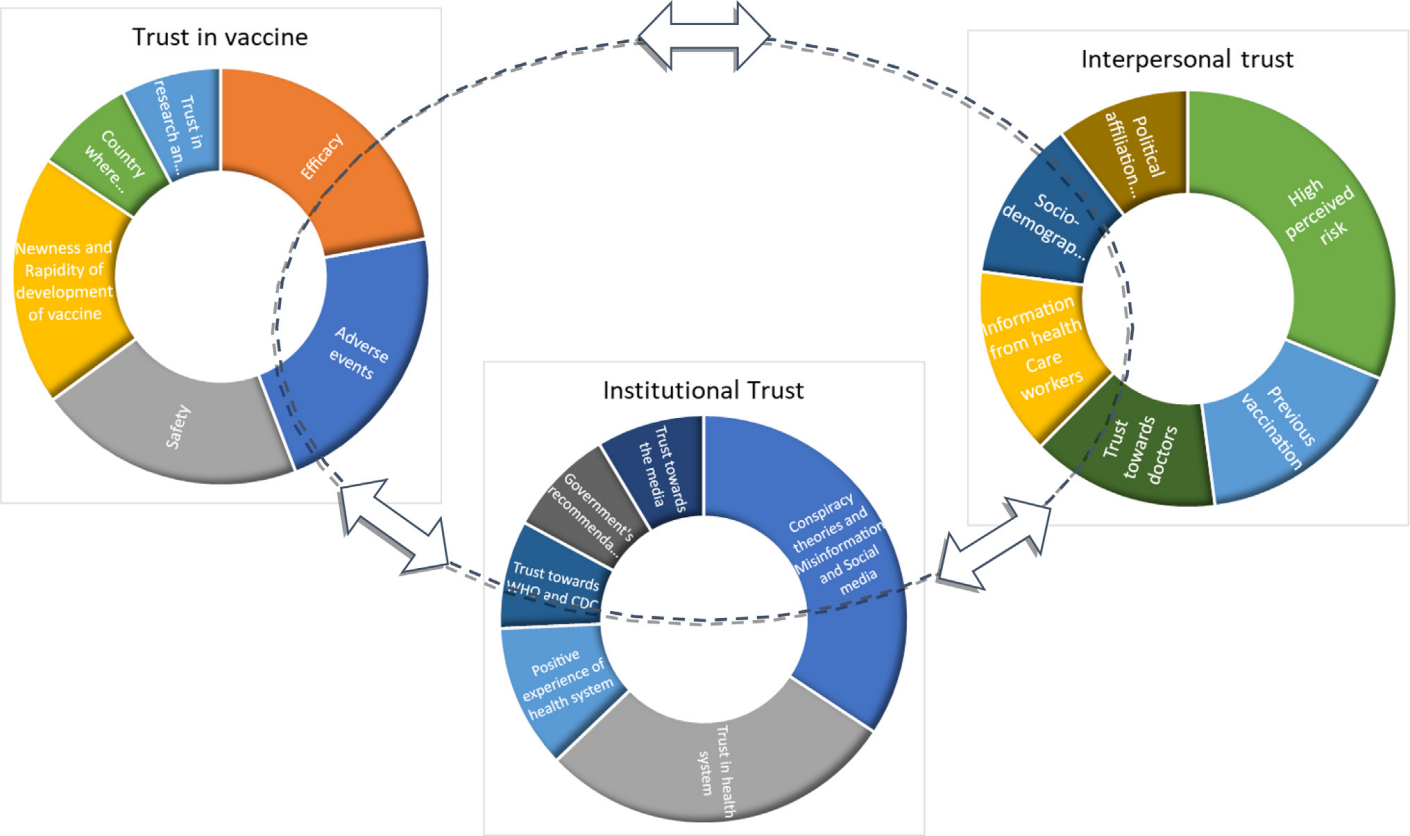
Types of trust and selected factors affecting trust in vaccine. The figure shows selected themes (based on the frequency) from the literature coded in NVivo. The themes were finally exported into Microsoft Excel to create the doughnut chart.

The majority of the literature reported how personal attributes and interpersonal trust affected the willingness to accept vaccination. Personal attributes in this review encompasses both socio-demographics (such as race, religion, and education) and behavioural characteristics (such as knowledge, perceptions and experience) which ultimately has impact on agency and vulnerability necessary for trust and relationship. The contributions made by institutional trust and trust in the vaccine were interwoven (Fig. 2). Except where trust was measured, it was described as a relational concept, dependent on factors embedded in local social, cultural, institutional, and personal attributes. The impact of trust was mostly reported in terms of willingness to accept COVID-19 vaccination.

### 3.2 Trust related to characteristics of vaccine (safety and quality related to vaccines)

Trust related to the characteristics of the COVID-19 vaccine was reported in most of the literature, and centred around efficacy, safety and associated adverse events [15], [24], [25], [26], [27], [28], [29], [30], [31], [32], [33], [34], [35], [36], [37], [38], [39], [40], [41], [42], [43], [44], [45], [46], [47], [48], [49], [50], [51]. Respondents were concerned about the limited experience with COVID-19 vaccines, which were at the beginning of 2021 invariably new and rapidly developed [15], [24], [26], [27], [29], [30], [31], [35], [36], [37], [39], [46], [47], [52], [53], [54]. Transparency related to vaccine development was found critical for trust in COVID vaccines and their uptake [30], [31], [36], [54], [55]. Specifically, the short time vaccine development has taken, the thoroughness of the vaccine trials, the estimated protective efficacy, and where the vaccine was tested were repeatedly found to be important factors in trust towards the vaccine and its acceptance [30], [31], [55]. Transparency related to vaccine development by commercial vaccine producers was perceived as critical to boost confidence in the vaccine by respondents in the UK [38]. Transparency in relation to the vaccine’s efficacy and side effects was perceived necessary to boost the trustworthiness of the vaccine and hence the willingness to get vaccinated in Japan [36].

Overall, trust in research and science was implied to be an integrated element of willingness to be vaccinated as an outcome [26], [33], [44], [46], [56], [57], [58], [59], [60], [61], [62], [63]. One of the factors that affected the trustworthiness of the vaccine was the country where the vaccine is developed [25], [55]. For instance, US citizens preferred vaccines made in the US over vaccines developed outside the country [27], [29], [31]. Similarly, Chinese respondents showed more trust towards domestic vaccines (increased willingness to be vaccinated with domestically produced vaccines) [25], [29]. Familiarity of the scientists producing the vaccine or at least regional representation in vaccine production was an important incentive to place trust for Cameroonians [55]. Vaccines which require vaccination regimens with fewer doses were likely to garner more trust and thus higher willingness to get vaccinated in China [28]. The longer the duration of protection, the higher was the trust towards the vaccine and the willingness to accept the vaccination [28].

### 3.3 Institutional trust

Trust in a vaccine was related to the trust in government and governmental institutions [44], [45], [60], [62], [63], [64], [65], [66]. The higher such institutional trust, the higher the trust in the vaccine and willingness to get vaccinated. Institutional trust was vulnerable to conspiracy theories and misinformation, especially through social media [15], [29], [40], [41], [46], [48], [50], [58], [60], [67], [68], [69], [70], [71]. People who believed in conspiracy theories and other forms of misinformation, or relied on information from social media were more likely to be suspicious about governmental institutions and more prone to distrust vaccines recommended by the state or its institutions than those who do not solely rely on such media for information. Trust in the health system of a country based on positive experiences or a good reputation affected the trust in vaccines provided by that health system [15], [26], [34], [35], [38], [43], [53], [63], [64], [72]. For instance, participants who had positive experiences with the National Health Services of the UK were enthusiastic about a COVID-19 vaccine [38]. As shown in a study in Italy, trust in the health system also originated from the perceived expertise and competence of healthcare workers and other representatives of the health system [68]. Specific recommendations from government were found to boost the populations’ confidence in and likelihood to accept vaccinations [25], [34], [44], [53], [66]. In contrast, historical grievances, racial injustice, systemic discrimination, unfair power, and status differences trigger suspicion and mistrust towards the institution, affecting vaccine uptake [44], [45], [60], [62], [63], [64], [73]. Among black HIV positive Americans, mistrust was also reported as a coping mechanism for historical or current dissatisfaction with treatment and services received from the government [73].

Trust in renowned national and international health organisations, such as WHO or the US Centers for Disease Control and Prevention (US-CDC), the state, and academia affect institutional trust and thus vaccine uptake [15], [27], [74]. A survey conducted among US adults showed that vaccines endorsed by the CDC and WHO were associated with an increase in acceptance compared to an endorsement by President Trump [27]. In early March 2021, the Oxford/AstraZeneca vaccine was briefly suspended in Europe due to fear of thromboembolic events, but the European Medicines Agency soon declared that the risk–benefit balance was positive and therefore endorsed the vaccine. Janssen’s COVID-19 vaccine is in the same class vaccines as the Oxford/AstraZeneca vaccine (viral vector vaccines) and its roll out in the US was also resumed after the Food and Drug Administration (FDA) and US-CDC reassured the public of its safety [75]. Nonetheless, pharmaceutical companies developing and producing vaccines, were perceived as having foremost financial interests in developing and marketing vaccines and ignoring the interests of the vaccine recipient [26], [53], [55].

The levels of trust in mainstream media, such as reputable television channels, newspapers and radio also correlated to the institutional trust because of their positionalities (for and against the government and the institution) which consequently affected the trust in the vaccine [15], [56], [66]. For instance, people who watched and trusted CNN were more likely to accept vaccines compared to followers of other channels such as Fox News [70]. There was wide recognition that media are biased in presenting the news and serving their interests, which may or may not align with the interests of the public. Based on the analysis of Spanish news outlets, media bias was also associated with the power difference between journalists and the state, whereby the state may impose pressure on media to influence the reporting of news [65]. Transparent health messaging was thought to be critical, especially when it comes to vaccine safety and efficacy in UK [38]. Any politicisation of COVID-19 as a disease or in relation to the vaccines negatively affected trust in the vaccines, as it triggered partisan views and political divisions in USA [15], [73].

### 3.4 Interpersonal trust and personal attributes of the potential vaccinee

Interpersonal trust was mostly dependent on an individual’s agency and vulnerability in accepting vaccines. Individual’s beliefs and perceptions thus have an impact on the interpersonal relationship and trust (e.g. with the health care workers) including trust towards the vaccine and the institution. In this section, personal attributes such as race, religion, and socio-demographic backgrounds’ impact on interpersonal trust are highlighted. The higher the perception of risk of becoming infected with COVID-19, and the severity of COVID-19 infections, the higher the motivation to get vaccinated [24], [28], [39], [45], [47], [53], [71], [72], [76], [77], [78]. One study from Kuwait showed that those who frequently informed themselves about the COVID-19 were more likely to accept the vaccine [79]. This was also true for those who had a higher level of health-related anxieties leading to a perception of increased risk of becoming getting COVID-19 [24], [68], [79]. In contrast, those who considered themselves at low risk for infection were less willing to accept a vaccination [25], [42], [44], [64], [80]. Perceived health benefits to self, family members and their societal context was also reported to be an important incentive to accept vaccination [24], [29], [37], [44], [45], [50], [77], [81], [82]. Willingness to be vaccinated was also shaped by their past experience of vaccinations [24], [26], [31], [39], [40], [42], [52], [54], [61], [63], [79], [83], [84], [85]. Political affiliations and inclinations were found to be critical in shaping trust and attitudes towards COVID-19 vaccines [34], [40], [56], [74], [86]. People with liberal political views and a democratic political inclination in US were more likely to accept a COVID-19 vaccination than conservatives voting republican [34], [40]. The socio-demographic characteristics of a person also shaped trust towards the vaccine, including age, gender, level of education, race, socio-economic status, religion, and marital status [15], [24], [25], [28], [34], [37], [40], [43], [45], [46], [47], [48], [49], [52], [57], [60], [62], [63], [64], [67], [71], [72], [79], [80], [81], [82], [87], [88], [89]. For instance, respondents lacking training in science subjects were less likely to trust the vaccine [43], [57], [60]. Knowledge of symptoms, transmission routes, prevention and control measures against COVID-19 was associated with a greater willingness to get vaccinated in a Greek population [71]. People who lacked specific knowledge about vaccines [40], [48], [83], [89], who had false beliefs related to the COVID-19 vaccine in the US [73], and those who did not trust the information from public health experts [30], [59] were less likely to trust in the benefits of vaccines. A study in Saudi Arabia showed that respondents with a high socio-economic status, male, older, and married were more likely to accept COVID-19 vaccinations than low-income, young, single and female respondents [72]. Race [15], [34], [37], [46], [49], [60], [62], [81], [86] and religion [87] had a significant impact on vaccine uptake. Residents of semi-urban and rural regions were more vaccine hesitant than their urban counterparts [43], [84], [86], [89]. In one UK-based study, Black, Asian, Chinese, mixed, or other ethnicities were almost three times more likely to reject the COVID-19 vaccine compared to Caucasian British [37]. Black Americans express significantly less trust in COVID-19 vaccines than their Caucasian counterparts [15], [34], [37], [46], [49], [60], [62], [81], [86]. Religiosity was associated with anti-science attitudes resulting in a reduced likelihood to be vaccinated in USA [87].

Healthcare workers in Israel and France were more likely to trust COVID-19 vaccines and more willing to accept vaccinations than people working in other sectors. Interestingly, nurses tended to be more hesitant than doctors [80], [90]. Various beliefs against modern science were reported to make significant impact in vaccine acceptance [42], [54], [73], [91]. For example, health care practitioners in Turkey and Austria who believed in alternative medicine and who practiced such medicine were less likely to trust modern science and vaccines [26], [92]. A study in Romania reported that those who believed in natural healing were more likely to look for alternatives to vaccine for prevention of disease, such as isolation, mask-wearing and maintaining hygiene [32]. Nurses in Palestine expressed a preference for natural immunity rather than acquiring immunity from a vaccine [48]. People fond of conspiracy theories [46], [48], [50], [60], [71], [91] perceived vaccines as unnecessary [42], [54] and held the belief that ‘vaccines do not work’ [42] and ‘vaccine can cause disease’ [46], [48]. They were more likely to reject vaccines and even go further by participating in vaccine distrust campaigns. Trust in COVID-19 vaccines appeared to be fragile. In the USA, even those who believed in the importance of scientific information were vulnerable to misinformation [56]. Globally, the adequacy of information about vaccines affected the trust and willingness to participate in vaccinations [54]. Studies emphasised the value of having an effective communication strategy by authorities rather than simply announcing the availability of vaccines to the population [35], [41], [44], [68], [85]. In Portugal, opportunities for dialogue, specifically for questions related to safety and efficacy of COVID-19 vaccines, were reported to mitigate vaccine hesitancy [63].

Peers, family members and health workers played a significant role in deciding whether or not to accept vaccination [24], [28], [30], [54]. For instance, a person’s trust in a doctor correlated with the trust in a vaccine and willingness to get vaccinated [30], [32], [38], [54], [60], [79], [91]. The impact of a venerated healthcare worker on promoting trust in vaccines was high in the USA [62], [74]. Information received from healthcare workers was more trusted and affected willingness to be vaccinated than any other sources of information [15], [30], [35], [39], [46], [50], [54], [73], [78], [91]. In China, when a family member, peer, or neighbour was vaccinated, the likelihood of accepting a vaccination also increased [28], [29]. There was also an increased likelihood to accept a COVID-19 vaccination if this was recommended by an employer [54].

Globally, the social, economic and health inequity in a minority population was found to trigger resentment against the privileged, elite and educated population who believe and promote science and vaccines [57]. Related to the current pandemic context, having experience of a prior epidemic reduced trust towards scientists and their work [57]. Based on a survey conducted among nurses, factors related to ease and feasibility of vaccination, such as accessibility, time it takes to get vaccinated, and cost were likely to undermine the trust in vaccine [42]. Based on the studies conducted in USA and Israel, people vulnerable to uncertainty, such as unemployment, lack of unemployment benefits and health insurance, were likely to seek protection from COVID-19 and thus may place trust in a vaccine [34], [90]. Free and voluntary vaccination increased vaccine acceptance compared to obligatory vaccination campaigns which can reverse trust [28], [47], [84]. The impact of COVID-19 outbreaks, such as inability to resume work and school, lockdowns, economic loses, and disrupted healthcare also increased the willingness to be vaccinated [25], [45], [54], [63].

## 4 Discussion

Many scientists, including vaccine developers and epidemiologists, seem to assume that the decision making for vaccination is an entirely rational process akin to a mental risk–benefit ratio. This is illustrated by the recent insistence by scientists that the ChAdOx-SARS-1 vaccine saves more lives than it will ever endanger [93]. Most non-scientists, and even some scientists, do not approach decision-making when it comes to vaccinations by calculating the risk–benefit ratio. This paradox is a normal phenomenon according to Daniel Kahneman [94], who outlined a dichotomy between two systems of thoughts relevant for decision making in the uptake of vaccine: a fast, emotional and unconscious thinking (system 1) and a slower, more infrequent, rational, and conscious thinking (system 2) [95]. Both systems are applied to decide whether or not to be vaccinated, although system 1) is often the resort for decision making. In the absence of a complete understanding of vaccines, which not many of us have, vaccine decisions are primarily based on prior experience and trust. Trust is a critical element for a person to decide whether or not to accept a vaccine, and is a combination of cognitive, affective and conative behaviour that is historically informed and culturally situated [6]. Willingness to take a vaccine (or absence of it) is deemed as outcomes arising out of trust of the individual in a vaccine; trust between an individual and an institution which procures it; and trust between an individual and the person who dispenses the vaccine [3]. The bulk of the literature reported how personal attributes and interpersonal trust affected overall trust towards the vaccine and willingness to accept the vaccine, and this accurately reflects the prominence of perception in contrast to decision making based on science.

Safety, quality and efficacy associated with vaccines are some of the rational concerns raised by potential vaccinees, nonetheless the science which could alleviate these concerns are neither comprehensible to general public nor reach to their daily living environments [96], [97]. Consequently, concerns around the vaccines are channelled through their peers, family members, neighbours and in recent years increasingly through social media outlets, which have been compared to a digital wildfire of information and misinformation [69]. The wide availability of inaccurate information via social media has been recognized as a parallel pandemic and has gained substantial attention as an ‘infodemic’ [98]. One of the essential implications is a need to fight against the misinformation unparallel to the past. Strategies to establish a responsive and legitimate information system more than ever before is critical to ensure that people can check their mis/conceptions, and have their queries accurately answered, including opportunities for dialogue [63]. In contrast to legitimate sources of information, opportunities to have non-judgemental dialogue with scientists in fact can help people to satisfy their queries. John Cook and colleagues’ concept of immunizing public against misinformation echoes a concept of psychological inoculation where a person is exposed to facts so that they can build ‘cognitive antibodies’ that can fight against the misinformation in future [99].

Safety and quality of vaccines are further threatened by the circulation of falsified and sub-standard vaccines which can jeopardize the effectiveness of vaccination programs, confuse and alarm communities and damage the public confidence in immunization programs [100]. Improving transparency and public engagement about the vaccine development process, safety, quality, and side effects can mitigate existing suspicion [30], [31], [54], [63]. Using creative and positive framings (positive emotions) to advocate on vaccine and its benefits are likely to promote public confidence. Safety and qualities of vaccines are often linked to the country of manufacture [25], [27], [29], [31], [54], [55]. Such associations may have arisen from increasing nationalism and parochialism which often demonstrate the poor understanding of vaccine production and procurement process, how vaccine quality is safeguarded by public health institutions such as agencies of the respective Ministries of Health and also international organisations such as the WHO. More broadly, such blinkered attitudes may hamper accommodating vaccine as a global public health tool rather than a mere national pride. Recent evidence around resurgence of COVID-19 and its variants due to unvaccinated population around, require deliberate strategies to steer ongoing discourses on global health solidarity, and vaccine equity [101].

Institutional trust is vulnerable to political conflict, historical injustices, and the ongoing grievances of populations and thus can manifest along the spectrum of non-participation, poor participation, resentment, rejection, and protests [15], [73]. The fact that the institutional trust is rooted and shaped by the historical and political treatment of population may offer solutions how such negative experience can be mitigated. Broader efforts addressing racial injustice, systemic discrimination, power and status discrimination are essential in establishing sustainable institutional trust [102].

At the same time, strategies to preserving the institutional trust associated with WHO, CDC-USA, and academic institutions need attention. Erosion of trust towards the reputed institutions can have catastrophic consequences as vaccines in general rely on recommendations by these institutions often referred to as ‘derivative merit trust’ [15], [27], [74]. Politicisation of science and vaccines has a direct impact on trust. Institutional trust continues to be threatened by the politicised rhetoric and populism that undermines experts in favour of their folk wisdom, narcissism and self-righteousness often explained as a cognitive bias namely the Dunning-Krueger effect [6]. Individuals with *a priori* distrust may simply hesitate to trust in science because of the conflicts between the science and their values [6], [21]. Individuals with *a priori* interests and biases require a tailored approach to reconcile new information with their values and beliefs rather than challenging their biases with the facts alone. Larson has highlighted an emphatic listening to ensure that their views are included, and respected before designing tailored and targeted interventions [103].

For instance, believers in natural healing tended to associate vaccines as ‘unnatural’ and a sign of weakness, as vaccines were seen to be an external, and unnecessary support to fight the disease [26], [32], [48]. Such cultural values should not be just undermined based on the scientific facts alone. For example, the ‘I immunise’ campaign from Western Australia where value-based reasoning was harmonised with the promotional messages related to vaccines resulted in increased coverage [6]. Population with contrasting beliefs and practice can be approached with strategies to integrate with their value system instead of outright rejection or undermining. Health care workers and public health agencies require intermediate approaches in dealing with the population with unscientific convictions and practices. Populations with strong anti-vaccine convictions and practice tend to form groups and protest against the science and technologies through conspiracy theories and distrust campaigns, thus further isolating themselves from mainstream thought. Past literature has explained such phenomena to be arising from increasing gaps between science and public, technological dominance in people’s daily lives, incomprehensibility of science and perceived inferiorities [6], [96]. Efforts to counter conspiracy theories that may seem to discount them or simply reject them may in fact trigger building of more theories in defence. It is therefore essential to understand these specific cohorts or communities and approach them through their respected leaders and the person they trust. Approaching respected figures who the community listens to and resort for advice e.g. religious/traditional leaders is critical to build trust[103].

## 5 Strengths and limitations

This qualitative review utilises systematic search of literature relevant to vaccine hesitancy and trust. The review attempts to address the research question by exploring the role of trust in vaccine uptake as opposed to other factors affecting vaccine uptake. Using a qualitative method to synthesise the evidence, this review includes a multitude of factors/themes that may have been overlooked in past reviews. The review attempted to explain the mechanisms underpinning the factors affecting trust and vaccine hesitancy. Although search strategies were targeted to explore literature around trust in vaccine; vaccine hesitancy and willingness to accept the vaccine were reported as proxy outcomes of trust towards the vaccines, thus the relationship between vaccine uptake (or its refusal) and trust can be precarious. Willingness to accept the vaccine (or vaccine hesitancy) may occur without having complete trust towards the vaccine, for instance when triggered by high perception of risk or when offered an irresistible incentive. At the same time, the perception of risk is a precursor during uncertainty, and trust entails ‘to be vulnerable to trustee during uncertainty’ [6]. This review attempts to extricate the trust when describing vaccine hesitancy or willingness to accept the vaccine, but under-reporting and lack of explicit reporting in the literature may have affected our thematic synthesis. In addition, using thematic synthesis to categorize wide spectrum of factors affecting vaccine hesitancy (refusal) into three broad categories of trust may have reduced the impact of individual factors. Limiting our search strategy by date and (English) language may have missed more recent literature, but our broader inclusion of literature on explanation helped us to achieve saturation. This review is based on cost-free availability of vaccine to protect against COVID-19. This may change in the near future and affect vaccine hesitancy. Future research could explore the relationship between trust and willingness to pay for the vaccine [104]. Although we have categorized ‘trust’ broadly into three general types in this review, there were no clear distinctions between these three types of trust in the literature and were intricately linked to each other.

## 6 Conclusions

We distinguish here three types of trust: (1) in the vaccine itself, (2) the institution distributing or administering the vaccine and (3) inter-personal trust. Each offers potential for approaches to nudge undecided people to receive vaccine; and vaccine refusers to revisit their decisions.

a. It is essential to build ‘vaccine trust’ by reminding people that the licensed COVID-19 vaccines are safe, protective and developed without cutting corners. Keeping people informed requires complete transparency about newly available information about vaccine. There is a risk that new safety concerns lead to temporary setbacks in trust building and vaccine uptake, but the alternatives have a disproportionately larger negative impact. A responsive and legitimate information system is critical to fight off the ‘infodemic’ and to ensure that people can check their conceptions, have their queries accurately answered and resolve conflicting messages from various sources including opportunities for dialogue. As the COVID-19 pandemic has demonstrated that it is not possible to build vaccine trust over a short time span. Long term information campaigns e.g. about the vaccine development process will be critical to prepare for future outbreaks and pandemics.
b. Continued support and endorsement for vaccine campaigns by reputed institutions are critical, which can garner ‘institutional trust’ towards the vaccine. This includes prominent figures, including the highest levels of local, regional, and national government continually encouraging the public to become vaccinated. Social, cultural, and religious institutions may play a more prominent role in promoting trust than government in different sub-populations and societies and as such, have to share or take over the role of government representatives in vaccine promotion.
c. Finally, ‘interpersonal trust’ and relationship play perhaps the most important part in building trust in vaccines. Populations around the world have significant interpersonal trust in their health care workers and more on those who they perceive to have shared identity. In general, existing trust and respect towards the health care workers can be a significant capital that could be utilized to boost the trust in vaccines. Engagement of health care workers in providing information and promoting vaccine confidence will help resolve the partisan attitude towards vaccine including dispelling myths and misinformation arising out of political rhetoric. Future empirical research around the correlation of trust and vaccine acceptance including mechanisms and processes that underpin it are essential in informing public, policy and stakeholder engagement and to promote vaccine coverage.

## CRediT authorship contribution statement

**Bipin Adhikari:** Conceptualization, Methodology, Data curation, Formal analysis, Software, Visualization, Writing - original draft, Writing - review & editing. **Phaik Yeong Cheah:** Methodology, Funding acquisition, Conceptualization, Writing - review & editing. **Lorenz von Seidlein:** Conceptualization, Methodology, Supervision, and manuscript review and editing.

## Declaration of Competing Interest

The authors declare that they have no known competing financial interests or personal relationships that could have appeared to influence the work reported in this paper.
