# Supplementary material for: Trust is the common denominator for COVID-19 vaccine acceptance: A literature review
Source: Vaccine X. 2022 Sep 29;12:100213. doi: 10.1016/j.jvacx.2022.100213 (PMC9536059; doi:10.1016/j.jvacx.2022.100213)
Supplement: Supplementary Data 1 [file mmc1.doc]

**Supplementary data 1**

**Search strategy**

**Date:** 5 February, 2021

**Pubmed (N=1362)**

(COVID-19 [MeSH Terms] OR COVID OR Corona OR SARS) AND (Vaccines [MeSH Terms] OR Vaccine OR Immunization [MeSH Terms] OR Immuni*) AND (Hesitan* OR Reluctan* OR Reject* OR Confid* OR Belief OR Percep* OR Trust* OR Faith OR Assur* OR Certain*)

**SCOPUS (n=825)**

TITLE-ABS-KEY (COVID-19 OR Corona OR SARS) AND (Vaccine OR Immuni*) AND TITLE-ABS-KEY (Hesitan* OR Reluctan* OR Reject* OR Confid* OR Belief OR Percep* OR Trust* OR Faith OR Assur* OR Certain*)

**Web of Science (n=484)**

TOPIC: (COVID-19 OR Corona OR SARS) AND (Vaccine OR Immuni*) AND TOPIC: (Hesitan* OR Reluctan* OR Reject* OR Confid* OR Belief OR Percep* OR Trust* OR Faith OR Assur* OR Certain*)

**Date:** 12 May 2021

**Pubmed (N=950)**

(COVID-19 [MeSH Terms] OR COVID OR Corona OR SARS) AND (Vaccines [MeSH Terms] OR Vaccine OR Immunization [MeSH Terms] OR Immuni*) AND (Hesitan* OR Reluctan* OR Reject* OR Confid* OR Belief OR Percep* OR Trust* OR Faith OR Assur* OR Certain*)

**SCOPUS (n=697)**

TITLE-ABS-KEY (COVID-19 OR Corona OR SARS) AND (Vaccine OR Immuni*) AND TITLE-ABS-KEY (Hesitan* OR Reluctan* OR Reject* OR Confid* OR Belief OR Percep* OR Trust* OR Faith OR Assur* OR Certain*)

**Web of Science (n=376)**

TOPIC: (COVID-19 OR Corona OR SARS) AND (Vaccine OR Immuni*) AND TOPIC: (Hesitan* OR Reluctan* OR Reject* OR Confid* OR Belief OR Percep* OR Trust* OR Faith OR Assur* OR Certain*)
